# Exploring the microbiome: Uncovering the link with lung cancer and implications for diagnosis and treatment

**DOI:** 10.1016/j.pccm.2023.08.003

**Published:** 2023-09-15

**Authors:** Junqi Yi, Juanjuan Xiang, Jingqun Tang

**Affiliations:** 1 Department of Thoracic Surgery, The Second Xiangya Hospital, Central South University, Changsha, Hunan 410013, China; 2 Hunan Key Laboratory of Early Diagnosis and Precise Treatment of Lung Cancer, The Second Xiangya Hospital, Central South University, Changsha, Hunan 410013, China; 3 NHC Key Laboratory of Carcinogenesis and the Key Laboratory of Carcinogenesis and Cancer Invasion of the Chinese Ministry of Education, Cancer Research Institute, Central South University, Changsha, Hunan 410028, China

**Keywords:** Lung cancer, Lung microbiome, Dysbiosis

## Abstract

Lung cancer is the leading cause of cancer-related deaths worldwide. Tobacco smoking and air pollution are believed to be responsible for more than 90% of lung cancers. Respiratory pathogens are also known to be associated with the initiation and development of lung cancer. Despite the fact that the bacterial biomass in the lungs is lower than that in the intestinal tract, emerging evidence indicates that the lung is colonized by a diverse array of microbes. However, there is limited knowledge regarding the role of dysbiosis of the lung microbiota in the progression of lung cancer. In this review, we summarize the current information about the relationship between the microbiome and lung cancer. The objective is to provide an overview of the core composition of the microbiota in lung cancer as well as the role of specific dysbiosis of the lung microbiota in the progression of lung cancer and treatment of the disease.

## Introduction

The lungs were previously considered to be sterile, but there is now increasing evidence to suggest the existence of a complex microbial community in the lungs, as in other parts of the body. Next-generation sequencing (NGS) technologies have provided a choice enabling high-throughput analysis of complex community. The total DNA and/or RNA is sequenced and characterized without prior cultivation. Amplicon sequencing and shotgun metagenomic sequencing are the two most commonly used culture-independent methods.^1^ Amplicon sequencing of the 16S ribosomal RNA (rRNA) gene or internal transcribed spacer rRNA is used to identify and compare bacteria or fungi from a given sample at the genus level. Shotgun metagenomic sequencing increases resolution and identifies species-level taxa. The community of microorganisms, including bacteria, archaea, eukaryotes, and viruses, is referred to as the microbiota. Recently, the terms “microbiome” and “microbiota” have been separated.^2^ Microbiota now refers to the entire collection of microorganisms in a specific niche whereas microbiome describes the entire ecologic community, including the sum of the microbes and their genomic elements, in a particular environment.^2^

Lung cancer is the leading cause of cancer-related mortality worldwide.^3^ So far, cigarette smoking is the leading risk factor. Exposure to secondhand smoke, radon, and asbestos also increases the risk of developing lung cancer. It has long been recognized that microbes affect the initiation and progression of cancer. Nejman *et al*^4^ profiled multiple types of cancer, including lung cancer, and found an abundance of bacteria in cancer cells and immune cells in the tumor mass. The intracellular microbiota has been described as a tumor component that can survive treatment with cell-impermeable antibiotics.^5^ The big data generated by NGS raise three key questions: Is there a core lung microbiota in patients with lung cancer? Do rare taxa, such as fungi, play as important a role in host physiology and pathology as taxa with high abundance? What is the mechanism that mediates the relationship between the lung microbiota and lung cancer? In this review, we summarize the current information about the relationship between the microbiome and lung cancer, aiming to provide quick answers to these key questions and describe the current limitations of microbiome research.

## Core lung microbiota in patients with lung cancer

The “core microbiota” is defined as a group of microbial taxa that remain stable over time and are shared by most humans.^6^ The core microbiota is considered crucial for the biological function of the host.^6^ Currently, two methods can be used to define the core microbiota in terms of microbial types and their functional importance. The first is based on the taxa that are shared by two or more microbial communities in a given host species or environment. The second is based on the taxa that are linked to essential biological functions.^6^^,^^7^ Identifying the core microbiome may assist in a better understanding of the maintenance of human health^7^[Table 1].

**Table 1:** Altered genera/species in the lung cancer microbiome.

**Categories**	**Genus/species**	**Sample**	**Methods**	**Reference**
Bacterium	*Granulicatella adiacens, Streptococcus viridans*	Sputum	Metagenomic sequence	37
Bacterium	*Granulicatella, Abiotrophia, Streptococcus*	Sputum	16S rRNA gene-sequencing	19
Bacterium	*Streptococcus, Neisseria*	Bronchial brushing samples	16S rRNA gene-sequencing	17
Bacterium	*Thermus*	Lung tissue samples	16S rRNA gene-sequencing	18
Bacterium	*Streptococcus, Veillonella*	Airway brushings	16S rRNA gene-sequencing	23
Bacterium	*Capnocytophaga, Veillonella*	Saliva samples	16S rRNA gene-sequencing	21
Bacterium	*Veillonella parvula*	Lung tissue samples	16S rRNA gene-sequencing	25
Bacterium	Gammaproteobacteria	Saliva samples	16S rRNA gene-sequencing	24
Bacterium	*Megasphaera, Veillonella*	Bronchoalveolar fluid	16S rRNA gene-sequencing	22
Bacterium	*Staphylococcus, Dialister*	Bronchial brushing samples, sputum samples	16S rRNA gene-sequencing	17, 19
Bacterium	*Legionella*	Lung tissue samples	16S rRNA gene sequencing	18
Bacterium	*Mycobacterium tuberculosis*	Bronchoalveolar fluid	Metagenomic sequencing	10
Fungus	*Malassezia*	Saliva samples	Massive sequencing	55
Virus	Human papillomavirus	Lung tissue samples	PCR	56, 57
Virus	Measles virus	Lung tissue samples	Immunohistochemistry	58

PCR: Polymerase chain reaction; rRNA: Ribosomal RNA.

### Taxa that are shared across patients with lung cancer

The composition of the core microbiota is typically determined by the level of amplicons. However, there is currently no consensus regarding what constitutes a healthy lung microbiota.^8^ While some experts have suggested that healthy lungs lack resident microbes, research indicates that the most abundant genera in the lungs of healthy individuals are *Prevotella, Streptococcus, Veillonella*, and *Neisseria*.^9^ Furthermore, shotgun sequencing has revealed that *Enterobacter* and *Mycobacterium* are prevalent genera in lung lavage fluid.^10^ The lungs also contain bacterial DNA from common oral taxa, which may reflect the aspiration of oral secretions.^11^

The commensal microbiota plays a crucial role in maintaining homeostasis and promoting the development of the host immune system.^12^ The immune system relies heavily on the appropriate composition of the microbiota. A well-balanced microbial community is of great importance in maintaining homeostasis. While there is still some debate over whether the shift in the lung microbiota should be referred to as dysbiosis,^8^ dysbiosis of the microbiota is typically characterized by a decrease in microbial diversity, changes in its functional composition, metabolic activities, and local distribution and an increase in proinflammatory species.^13^ Despite variations in the enrichment of taxa across studies, decreased diversity has been associated with an increased risk of lung cancer.^14^, ^15^, ^16^

Several studies have investigated the presence of specific taxa in patients with lung cancer. *Staphylococcus* and *Legionella* were found to be less abundant in these patients, suggesting a protective role in the development of lung cancer.^17^^,^ ^18^ Lobectomy alveolar lavage samples showed that *Alloprevotella rava* and *Haemophilus paraphrohaemolyticus* were less abundant in patients with non-small cell lung cancer (NSCLC) than in controls.^10^ Various studies have reported that *Granulicatella, Abiotrophia, Streptococcus, Thermus, Veillonella, Capnocytophaga, Actinomycetales, Variovorax, Gammaproteobacteria, Herbaspirillum, Sphingomonadaceae*, and *Megasphaera,* among others at the genus level, are more abundant in bronchoalveolar lavage fluid (BALF) samples, bronchoscopic-protected specimen brushing, and sputum samples from patients with lung cancer compared to those from normal controls.^15^^,^^17^^,^^19^, ^20^, ^21^, ^22^, ^23^, ^24^

Comparison of lower airway samples from patients with stage IIIB–IV NSCLC with those from patients with stage I–IIIA NSCLC revealed that *Moraxella, Fusobacterium, Pseudomonas*, and *Haemophilus* were enriched in the advanced stages, whereas *Actinomycetales* was enriched in the earlier stages.^25^ After multivariate analysis adjusted for tumor-node-metastasis stage, smoking status, and type of treatment, enrichment of *Streptococcus, Prevotella, Lactobacillus, Gemella*, and *Veillonella* was found to be associated with a poor prognosis.^25^ The variation in results between studies may reflect the use of different sampling methods and other confounding factors, such as lifestyle, diet, and environment. However, the contradictory findings regarding the enrichment of taxa in the lung microbiota make it difficult to identify the actual species or strains that have deleterious or preventive roles in carcinogenesis. The heterogeneity of the results suggests that the core microbiome may be dynamic, differentially distributed, and functional, comprised of functional gene clusters rather than individual taxa.^7^

### Upper and lower airways

The tracheobronchial tree contains 20–25 generations. The microbial composition differs across different regions of the airway.^10^ The composition of the lung microbiome is influenced by the migration of microbes in and out of the airways, the elimination of microbes from the airways, and the growth of community members.^26^ The lung microbiota is enriched with oral-related taxa, suggesting aspiration of oral secretions into the upper and lower airways. Microbial diversity is the highest in the upper airway and decreases toward the lower airway in patients with lung cancer.^10^^,^^27^ Bacterial concentration and diversity are significantly reduced in the lower airway as a result of bacterial migration rates and removal by the mucociliary and immune systems.^27^ Various samples, including BALF, sputum, saliva, and lung biopsy, are used to evaluate the composition of the lung microbiome in relation to lung cancer. Some studies have investigated the composition of the microbiota according to the sampling method used. BALF collected from lobectomy samples, which represent the lower airway, contains more *Mycobacterium tuberculosis* (*M. tuberculosis*), *Streptococcus pneumoniae,* and other microbes whereas BALF collected from bronchoscopy samples contains a higher abundance of *Porphyromonas, Veillonella, Prevelar,* and other microbes.^10^ Bronchial washing fluid collected from patients with lung cancer was found to contain a higher abundance of Proteobacteria than sputum samples, and the microbiome of the bronchial washing fluid samples was more similar to that of lung cancer tissues.^28^

### Co-evolution of the microbiota with the host environment

It has long been realized that environmental exposures affect lung health. The microbiota and its host environment have co-evolved over millions of years, resulting in a complex and dynamic relationship. The host environment provides the microbiota with nutrients and a place to live, while the microbiota contributes to various physiological processes, such as digestion and immunity. Over time, the host environment has shaped the composition of the microbiota and vice versa.

The co-evolution of the microbiota and host environment has important implications for health and disease. Recent investigations have highlighted the association between specific environmental microbiomes and respiratory health.^29^ Mucosal surfaces and skin exposed to the external environment are colonized by a vast number of microbes.^12^^,^^30^ In this regard, the host-associated core microbiome is often referred to as key microbes in view of their spatial distribution, temporal stability, or ecological influence as well as their contribution to the function and fitness of the host.^6^

Several factors, including cigarette smoke, asbestos, and particulate matter smaller than 2.5 µm in diameter, increase the likelihood of developing lung cancer. Smoking causes a variety of airway diseases and affects the upper and lower airway microbiome in healthy smokers, ex-smokers, and patients with a smoking history.^31^ Studies have shown that smoking alters the microbial community in the lungs, leading to an increase in potentially harmful bacteria and a decrease in beneficial bacteria. This shift in the microbial community can have negative effects on lung health and may contribute to the development of lung cancer. Firmicutes and Actinobacteria have a higher relative abundance, whereas Proteobacteria have a lower relative abundance in smokers than in never-smokers. *Streptococcus* and *Veillonella,* which are the most common genera in the lungs, do not show a significant difference in individuals with a smoking history; *Staphylococcus epidermidis* may explain the higher abundance of Firmicutes in smokers, especially those who have smoked for a long time.^31^ The anaerobic bacterium *Atopobium*, which is a genus of Actinobacteria, shows a significant positive correlation with a number of years of smoking and with nicotine levels. However, *Betaproteobacteria*, a class of Gram-negative bacteria belonging to the phylum Proteobacteria, is less abundant in smokers.^31^ *Acinetobacter*, which is a genus of Gammaproteobacteria, is significantly more abundant in smokers and ex-smokers than in never-smokers.^31^ The Gram-negative bacteria *Leptotrichia* and *Neisseria* show significant negative correlations with years of smoking, smoking nicotine levels, and smoking intensity.^31^

Increased alpha diversity is associated with the use of smoky coal compared to clean fuel, and the presence of livestock in the home.^14^ Subjects with lower microbiota alpha diversity have an increased risk of lung cancer compared with those with higher alpha diversity.^32^ A greater abundance of *Spirochaetes* and *Bacteroidetes* is associated with a decreased risk of lung cancer and greater abundances of Bacilli class and Lactobacillales order are associated with an increased risk of lung cancer.^32^

However, the composition of the lung microbiota can also influence the host environment. Studies have found that certain bacteria in the lungs can produce metabolites that affect the immune response and inflammation in the respiratory system.^33^ These metabolites can either promote or suppress inflammation depending on the specific bacteria involved, which suggests that the composition of the lung microbiota can play a role in shaping the host's immune response and overall lung health.

### Microbiota in co-occurrence networks

Microorganisms within communities establish various relationships, including commensalism, synergism, competition, and parasitism. Cooperative interactions, such as commensalism, enhance biological fitness and facilitate coevolution of species. On the other hand, negative interactions, such as competition and parasitism, reflect the selection pressure from certain species on other species.^34^ Microbial co-occurrence networks can be used to explore connections in microbial communities, allowing for the prediction of hub species and potential species interactions. Co-occurrence networks may also be indicative of environmental adaption and habitat preference.^34^ The genus-level correlation network plots revealed significant interactions between different genera, with *Prevotella, Alloprevotella, Veillonella, Pseudomonas*, and *Rhodococcus* being the five most significantly associated genera in the network. The first three genera were more likely to co-occur in the lung cancer group and the latter two in the group with benign lung disease.^35^ Research has estimated co-occurrence probabilities between taxa and the host transcripts and found that the *Veillonella, Prevotella*, and *Streptococcus* genera have a high probability in patients with stage IIIB–IV NSCLC and that the *Flavobacterium* genus has a high probability in patients with stage I–IIIA disease.^25^ In other research, more connections were found between the genus *Streptococcus* and other genera, including *Porphyromonas, Prevotella, Capnocytophaga, Veillonella, Atopobium, Actinomyces, Rothia*, and *Granulicatella*, in patients with advanced NSCLC. However, other researchers have found that the co-occurrence of *Actinomyces* and the *Granulicatella, Veillonella, Prevotella,* and *Streptococcus* genera is exclusive to patients with early NSCLC.^36^ A pilot metagenomic sequencing study demonstrated the co-occurrence of *Granulicatella adiacens* and *Enterococcus sp. 130, Streptococcus intermedius, Escherichia coli* (*E. coli*)*, Streptococcus viridans, Acinetobacter junii,* and *Streptococcus sp. 6* in sputum samples from patients with lung cancer.^37^

### Taxa that are linked to essential biological functions

**Fig. 1:**
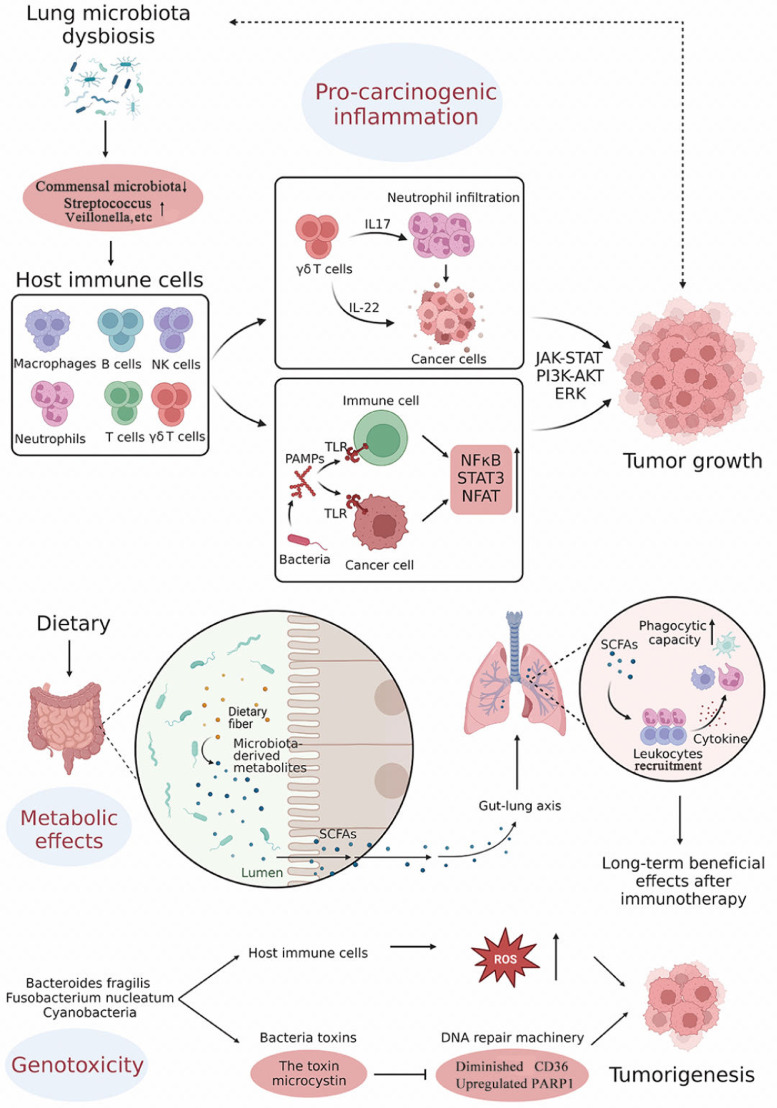
Biological functions of the lung microbiome. There are three potential carcinogenic functions that may be linked with the lung microbiome: (1) pro-carcinogenic inflammation; (2) metabolic effects, and (3) genotoxicity. AKT: Protein kinase B; ERK: Extracellular signal-regulated kinase; IL: Interleukin; JAK: Janus kinase; NFAT: Nuclear factors of activated T cells; NFκB: Nuclear factor kappa B; NK: Natural killer; PAMPs: Pathogen-associated molecular patterns; PARP1: Poly (ADP-ribose) polymerase 1; PI3K: Phosphoinositide 3-kinase; ROS: Reactive oxygen sepcies; SCFAs: Short-chain fatty acids; STAT: Signal transducer and activator of transcription; TLR: Toll-like receptor.

Given the lack of consensus regarding the composition of the core microbiome, researchers have focused on the genes, functional pathways, and metabolic profiles common to microbial communities in specific environments.^7^ There are three potential carcinogenic functions that may be linked with the lung microbiome: pro-carcinogenic inflammation, metabolic effects, and genotoxicity^38^ [Fig. 1].

It has long been known that there is a causal relationship between inflammation and cancer. The abundance of bacteria present in healthy lungs is low because the immune cells in the lungs control the balance between immune tolerance and immune clearance.^39^^,^ ^40^ The shift from Gammaproteobacteria and Firmicutes toward Bacteroidetes during the first 2 weeks of life is associated with the accumulation of a PD-L1-dependent T-regulatory cell population that promotes tolerance to allergen challenge.^41^ Innate immune cells including monocytes/macrophages, γδ T-cells, natural killer T-cells, innate lymphoid cells, and adaptive immune cells including specific subsets of resident memory B-cells and T-cells are involved in the microbiota-associated immune system in the lung.^20^ The altered bacterial diversity and altered composition of the lung microbiota can activate tissue-resident lymphocytes to establish a pro-tumorigenic microenvironment.^42^ The major tissue-resident T-cell population consists of γδ T-cells, which display innate-like characteristics and protect against infection in the lungs. Application of antibiotics impairs the induction of functional γδ T-cells and the production of interleukin (IL)-17, leading to accelerated pulmonary metastasis, emphasizing the highly context-specific microbiome-immune interaction.^43^ However, lung-resident γδ T-cells have an unappreciated role in promoting tumorigenesis in the lung. In this context, the lung microbiota provokes cancer-associated inflammation via lung-resident γδ T-cells producing IL-17 and infiltration of neutrophils, and germ-free or antibiotic-treated mice are significantly protected from the development of lung cancer.^15^ Application of antibiotics also leads to increased activation of anti-tumoral natural killer cells and T-cells, promoting an immune response in the tumor microenvironment.^44^ Pattern recognition receptors (PRRs) play an important role in the immune response by recognizing molecules frequently found in pathogens, namely, pathogen-associated molecular patterns. Toll-like receptors (TLRs) are expressed in both immune cells and cancer cells. TLRs are considered to act as a double-edged sword in the progression of cancer.^45^ Microbiome-mediated activation of TLRs promotes the progression of lung cancer through pro-inflammatory and anti-inflammatory factors.^46^ These detrimental effects occur mainly via activation of nuclear factor kappa-B (NFκB), signal transducer and activator of transcription (STAT) 3, or nuclear factors of activated T cells (NFAT).^47^ Enrichment of *Streptococcus* and *Veillonella* in the lower airways of patients with lung cancer has been associated with upregulation of the extracellular signal-regulated kinase (ERK) and phosphoinositide 3-kinase (PI3K) signaling pathways.^23^ In one study, the taxa with the highest abundance of *Veillonella parvula* was recognized to be strongly associated with cell adhesion molecules, IL-17, cytokines, and activation of the Janus kinases (JAK)-STAT and PI3K-protein kinase B (AKT) signaling pathways. In that study, the role of *Veillonella parvula* in the activation of pathways and upregulation of cytokines such as IL-17 and IL-6 was confirmed in KP (Kras^LSL-G12D/+^;p53^fl/fl^) mice.^25^

The metabolomics of the microbiota provides the link between the microbiota and its functions. Some microbiota-derived metabolites, including short-chain fatty acids (SCFAs), branched-chain amino acids, trimethylamine N-oxide, tryptophan, and indole derivatives, have been implicated in the progression of cancer and may serve as potential diagnostic biomarkers and therapeutic targets.^48^ Depletion of serum levels of branched-chain amino acids, methylhistamine, and vitamins associated with decreased abundance of *Prevotella copri* and *Lactobacillus gasseri* is thought to be linked with cancer-associated cachexia.^49^ SCFAs are small carboxylic acids, of which acetic acid, propionic acid, and butyric acid are the most abundant in the gut.^50^ Production of SCFAs by the gut microbiota affects the immune status of the lung via the gut–lung axis.^51^ An association between SCFAs and the abundance of *Alistipes* and *Bacteroides* in the gut microbiota has been reported in patients with lung cancer. The link between increased lung function and enriched abundance of certain genera, such as the *Veillonella* and *Lactobacillus,* demonstrates that the treatment outcome might be improved by butyrate-producing species.^52^ A small study found that gut microbiota metabolites were closely associated with the response to immunotherapy in patients with NSCLC. 2-Pentanone (a ketone) and tridecane (an alkane) were significantly associated with a poor response to immunotherapy, whereas SCFAs (i.e., propionate and butyrate), lysine, and nicotinic acid were significantly associated with long-term beneficial effects after immunotherapy.^53^ SCFAs regulate the functions of immune cells by recruiting leukocytes, particularly neutrophils, modulating the production of inflammatory mediators, and affecting the phagocytic capacity of phagocytes.^54^

The genotoxicity of microbes has been widely acknowledged in viral infection as a result of the integration of the virus genome into the host genome. However, a link between bacteria and genotoxicity arises from DNA damage. Bacteria such as *Bacteroides fragilis* and *Fusobacterium nucleatum* can cause DNA damage via reactive oxygen species derived from host immune cells or directly via bacterial toxins.^47^ Bacterial toxins such as cytolethal distending toxin, *Bacteroides fragilis* toxin, and cytotoxic necrotizing factor-1 can damage DNA repair machinery and promote tumorigenesis. Microcystin, a toxin from *Cyanobacteria*, has been associated with diminished CD36, upregulation of poly (ADP-ribose) polymerase 1 levels, and progression of lung cancer.^46^

## Important components: rare taxa

Rare taxa refer to microorganisms or species that have a low abundance (less than 1%) in a particular environment or ecosystem and are often of interest to researchers because they may play important roles in the functioning of the ecosystem. Microbiome studies sometimes focus on the behavior of a specific group of microbiota, such as a mycobiome, or the behavior of a specific opportunistic pathogen. The interactions between groups or between groups and specific species have diverse consequences^55^, ^56^, ^57^, ^58^ [Table 1].

### Interplay of microbiota and pathogens in lung cancer

The intricate interactions between the host, pathogens, and microbiota determine the outcome of an infection. Members of the microbiota exist in a delicate ecological balance with one another.^59^ Microbe–microbe interactions enable the persistence of some species and the exclusion of others. In addition to niche competition, the microbiota can produce molecules such as bacteriocins that inhibit pathogens.^59^ For example, *Ruminococcus obeum* restricts *Vibrio cholerae* by expressing luxS.^60^ Enteric pathogens and pro-inflammatory bacteria are more frequently found in lung cancer tissues than in adjacent healthy tissues.^61^ In our previous study, we found a high abundance of *M. tuberculosis* in hospitalized patients, including those with lung cancer.^10^ Dysbiosis of the microbiota results in increased early colonization of the lungs by *M. tuberculosis*, possibly because of the reduced number of mucosal-associated invariant T-cells, which are dependent on a balanced host microbiota.^62^ The relationship between *M. tuberculosis* and lung cancer is complex, considering that tuberculosis can increase the risk of lung cancer by prolonged pulmonary inflammation, fibrosis, and genetic alterations.^63^

### The mycobiota and lung cancer

Fungi are more complex than bacteria and viruses. As eukaryotes, fungi have a structure similar to that of animals. Commensal fungi exist in various parts of the body, including the oral cavity, gastrointestinal tract, and vagina. Interactions between fungi and bacteria play a key role in the maintenance of ecosystems. Alterations in the mycobiome have been observed in many types of cancer in humans, including colorectal cancer, pancreatic ductal adenocarcinoma (PDAC), gastric cancer, and lung cancer.^10^^,^^64^, ^65^, ^66^ Reduced relative abundance of *Mucoromycota* and increased relative abundances of *Sordariomycetes, Saccharomycetaceae*, and *Malassezia* are observed in the composition of the gut microbiota in patients with colorectal cancer and those with PDAC.^67^ *Malassezia* species have been found to be elevated in colorectal cancer and PDAC, and their abundance shows good accuracy in differentiating patients with colorectal cancer and PDAC from healthy controls.^65^ Fungal genera that constitute the pulmonary mycobiota include *Candida, Malassezia, Neosartorya, Saccharomyces*, and *Aspergillus*.^68^ *Cladosporium* species, *Aspergillus* species, and *Penicillium* species dominate the fungal genera in the oral and nasal cavities.^69^ The presence of *Fusarium* and *Cryptococcus* in the lungs but not in the oral cavity of healthy individuals suggests that the fungal communities harbored in the lower airways are distinct from those harbored in the upper airways. Inhaled environmental fungi can disrupt fungal homeostasis in the lungs. Inhaled fungi, such as *Aspergillus, Cladosporium*, and *Penicillium,* are a well-established cause of asthma.^70^ Analysis of the central lung cancer mycobiome revealed a higher level of *Malassezia* in patients with lung cancer and higher levels of *Candida* in controls.^55^

### The virome and lung cancer

Increasing attention is being paid to the role of the global virome in health and disease. Metagenomic DNA sequencing has revealed the composition of the microbiota, including the virome.^71^ Viruses are capable of settling in a host cell and are minimally influenced by environmental changes. Viruses can be inherited when germline cells are infected. Human endogenous retroviruses are the most abundant viral elements integrated into the human genome. Human herpesvirus 6, cytomegalovirus, and Epstein–Barr virus establish long-term latency in the vast majority of humans.^72^ Anelloviridae and Redondoviridae are predominant, with sporadic detection of herpesviruses, papillomavirus, and retroviruses. The levels of these viruses are higher in the airways of critically ill patients than in those of healthy individuals. However, the effects of these viruses on the host and disease remain unclear.^73^ Human papillomavirus and the measles virus are the two common viruses associated with lung cancer.^56^, ^57^, ^58^ However, the roles of viruses in lung cancer require further investigation.

### Intracellular lung microbiota and lung cancer

Bacteria can be classified into two distinct groups: extracellular bacteria, which proliferate in the extracellular environment and are enriched in body fluids, and intracellular bacteria, which infect and replicate inside host cells.^74^ Intracellular bacteria can be further divided into facultative intracellular bacteria, which replicate either inside eukaryotic host cells or in an environmental niche, and obligate intracellular bacteria, which replicate exclusively inside eukaryotic host cells.^74^ The immune system provides a host defense against pathogens. Upon bacterial infection, PRRs located on the cell surface of phagosomal membranes or the cytosol of innate immune cells^75^ activate monocytes and macrophages. The binding of the outer membrane component of bacteria to PRRs activates PRR signaling, which increases the phagocytosis of bacteria by immune cells.^75^ PRRs such as TLRs play a crucial role in the innate immune system by recognizing pathogen-associated molecular patterns of microbes. In contrast with TLRs that mediate extracellular recognition of microbes, nucleotide oligomerization domain (NOD)-like receptors initiate an innate immune response by sensing pathogens in the cytosol.^76^

Intratumoral bacteria are mostly intracellular and can be found in both cancer and immune cells.^4^ In cancer cells and CD45-positive leukocytes, bacterial 16S rRNA is predominantly detected in the cytoplasm. Lipopolysaccharide, the major component of the cell wall in gram-negative bacteria, is present in both the cytoplasm and nucleus of cancer cells. However, the major cell wall component of gram-positive bacteria, lipoteichoic acid, is found almost exclusively in macrophages. Lipoteichoic acid-positive bacteria are rarely detected in cancer cells or CD45^+^/CD68^−^ immune cells. However, fluorescence *in situ* hybridization has shown that 16S rRNA is found infrequently in macrophages, suggesting that lipopolysaccharide and lipoteichoic acid can persist in these cells long after the phagocytosis of live bacteria.^4^

Intracellular bacteria, such as *Chlamydophila pneumoniae* (*C. pneumoniae*)*, M. tuberculosis, Cryptococcus* species and *Helicobacter pylori*, have been associated with lung cancer.^77^ Specific *C. pneumoniae* immunoglobulin A (IgA) antibodies were found significantly more often in patients with lung cancer than in controls.^78^ Metabolic functions encoded by intratumoral bacteria are linked with clinical features of certain tumor subtypes. Effective growth and replication of intracellular pathogens depend on energy and nutrients from host cells.^79^ Infection of mammalian cells with *C. pneumoniae, M. tuberculosis, Legionella pneumophila,* or other microbes can result in altered metabolism, such as an increment in glucose uptake and/or glycolysis.^79^ The Warburg effect, which is observed in proliferating cancer cells, is also observed in immune cells.^75^ The immune function of macrophages is determined by their polarization, whereby M1 macrophages have pro-inflammatory and tumoricidal properties and M2 macrophages have anti-inflammatory and tumor-promoting properties.^75^ M1 macrophages show increased glycolysis and decreased oxidative phosphorylation, whereas M2 macrophages show higher oxidative phosphorylation, mitochondrial fatty acid oxidation, and α-ketoglutaric acid (α-KG). *M. tuberculosis* infection causes a Warburg-like shift by upregulation of multiple glycolytic enzymes and glucose uptake transporters and downregulation of enzymes participating in oxidative phosphorylation and the tricarboxylic acid cycle.^80^ *M. tuberculosis* causes an increase in glucose uptake in infected cells, which can be used as a surrogate marker for Warburg metabolism through fludeoxyglucose (FDG)-positron emission tomography.^75^ Metabolic reprogramming of macrophages, which is characterized by upregulation of glucose uptake and synthesis of large lipid bodies derived from glycolytic intermediates, feeds intracellular *M. tuberculosis*.^75^

The presence of intracellular bacteria in patients with lung cancer is linked to their capability to degrade chemicals found in cigarette smoke, such as nicotine, anthranilate, toluene, and phenol.^4^ Metabolic MetaCyc pathways in the microbiome are responsible for the degradation of these chemicals.^4^ High levels of the cigarette smoke metabolites provide a preferred niche for bacteria that can use these metabolites.^4^ In comparison with non-smokers with NSCLC, smokers with lung tumors have more bacteria with enriched functions, including biosynthesis of cytidine monophosphate (CMP)-sugar and degradation of aromatic compounds, 2-aminobenzoate, choline, and phenolic compounds.^4^

## Gut microbiota, gut–lung axis, and lung cancer

The human gut microbiota plays a crucial role in the health of the host. The effect of the gut microbiota is not limited to the gut but extends to systemic compartments and distant organs.^81^

### Gut microbiota and lung cancer

Studies have explored the impact of gut microbiota on extra-gastrointestinal tumors, including lung cancer. Researchers have found that the composition of the gut microbiota in patients with lung cancer is different from that in healthy individuals, with higher levels of *Bacteroides, Veillonella*, and *Fusobacterium* in fecal samples but lower levels of *Escherichia, Shigella, Kluyvera, Faecalibacterium, Enterobacter*, and *Dialister*.^82^ The oral, lung, and gut microbiomes can communicate with each other in a direct or indirect manner.^83^ The presence of a “gut–lung axis” is believed to partially explain the role of the gut microbiota in the progression of lung cancer.

### Gut–lung axis and lung cancer

The gut–lung axis is a bidirectional communication network between the gut and the lungs that is connected through various mechanisms, including the circulation of immune cells, cytokines, microbial metabolites, and microbes. The respiratory tract and gut may communicate with each other by micro-aspiration and inhalation.^83^ The gut–lung axis partially arises from shared mucosal tracts in conjunction with lymphoid tissues and the migration of immune cells. Translocating bacteria, cell wall fragments, and protein fractions can escape immune defense and travel into the circulatory system, gaining access to the pulmonary circulation. This may lead to the activation of dendritic cells and macrophages in the lungs.^84^ Gut-derived lipopolysaccharide and SCFAs produced by the gut microbiota can also be transmitted to the lungs and affect pulmonary function directly or have an indirect effect via stimulation of the gut/circulating immune system.^51^^,^ ^84^

**Fig. 2:**
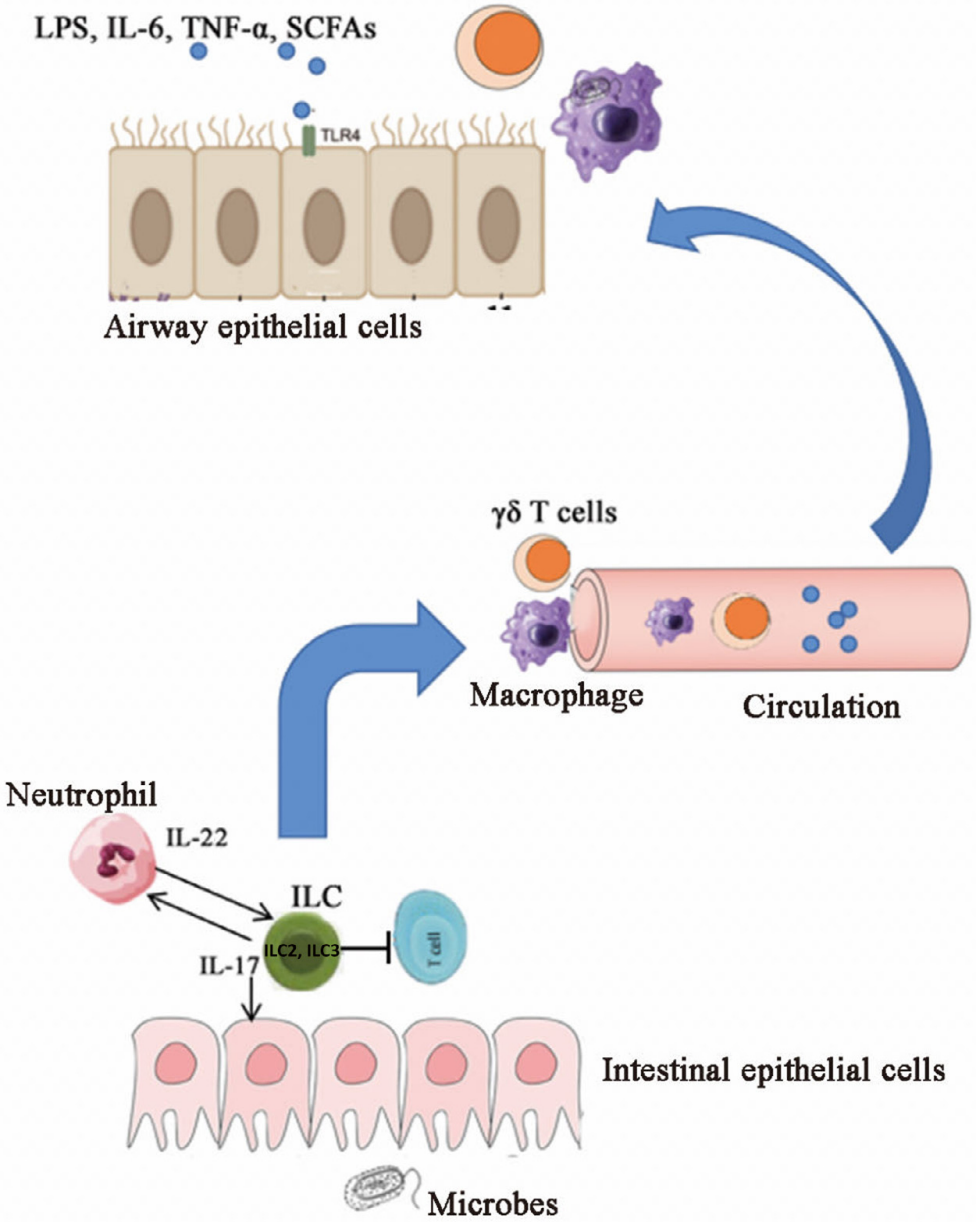
The gut microbiota and the gut–lung axis. Complex crosstalk occurs between the immune systems of the lung and gut. Gut immune cells, macrophages, and γδ T-cells in the gut can traffic to the lungs. Innate lymphoid cells (ILC2 and ILC3) that produce IL-17 and IL-22 recruit neutrophils and suppress commensal-specific T-cells. Innate lymphoid cells migrate from the gut to the lungs driven by IL-33-CXCL16 signaling. Gut-derived lipopolysaccharide, IL-6, tumor necrosis factor-α, and short-chain fatty acids migrate to the lungs and activate inflammation signals via TLR 4. IL: Interleukin; ILC: Innate lymphoid cells; LPS: Lipopolysaccharide; TLR: Toll-like receptor; SCFAs: Short-chain fatty acids; TNF-α: Tumor necrosis factor-α.

The gut microbiota plays a role in directing the migration of immune cells. For example, the IL-33-CXC-chemokine ligand 16 (CXCL16) signaling induced by Proteobacteria facilitates the migration of innate lymphoid cells (ILC2) from the gut to the lungs [Fig. 2]. *Enterobacter* has been detected in the lung microbiome, and its abundance is negatively associated with smoking.^10^^,^^85^ Translocation of the gut microbiome, such as Bacteroidetes and Enterobacteriaceae, to the lungs has been found in patients with pulmonary diseases,^86^ but the IL-22/STAT3 signaling plays a crucial role in safeguarding the integrity of the gut barrier and preventing such translocation.^87^ In addition to lung disease, cigarette smoking is also a major risk factor for intestinal disorders, partly because of impaired mucosal immune responses triggered by an altered microbiome.^88^ The toxins found in cigarettes, such as nicotine, acrolein, hydrogen sulfide, cadmium, lead, and arsenic, increase the relative abundance of Firmicutes and Proteobacteria and decrease the relative abundance of Bacteroidetes. Gram-negative bacteria such as *E. coli, Salmonella typhimurium, Enterococcus faecalis*, Lactobacillaceae, and Lachnospiraceae, are decreased in the intestinal microbiota upon exposure to toxic compounds in tobacco.^88^

### Mucosal immunity and gut–lung axis

Mucosal immunity, a type of immune response that occurs at the mucosal surface, plays an important role in the gut–lung axis. Immune cells, including gut-associated lymphoid tissue, mucosa-associated lymphoid tissue, bronchus-associated lymphoid tissue, lung-associated lymphoid tissue, and airway epithelial cells (AECs), play an important role in regulating the immune response in the gut and lungs. The mucosal surface of the lung is colonized by the microbiota, which provokes the inflammation associated with lung adenocarcinoma by activating lung-resident γδ T-cells that produce IL-17 and other effector molecules.^15^ Treatment with antibiotics that partially eliminate commensal bacteria in the lungs but not fecal bacteria reduces the tumor burden.^15^ The “common mucosal response” exerted by the gut microbiota and its metabolites activates the remote immune response in the lungs.^89^ The immune system can be divided into a series of functional anatomical compartments.^90^ In addition to the innate and adaptive immune responses, which react to antigens in the peripheral lymphoid system, a second compartment of the adaptive immune system, namely, the mucosal immune system (mucosa-associated lymphoid tissue), plays a critical role in host–microbe interactions.^90^^,^^91^ Th17 cells produce IL-17, IL-21, and IL-22, which are critical for defense against bacterial, fungal, and viral infections at the mucosal surface.^92^ The adaptive immune response triggered by microbes involves B-cells, which mediate gut homeostasis by producing secretory IgA antibodies that are responsive to commensals.^93^ Innate responses are mediated not only by blood cells such as neutrophils and macrophages but also by intestinal and lung epithelial cells that transmit signals in the mucosa.^94^

AECs are the most abundant type of cell in the lungs and contribute directly or indirectly to defense via the epithelium and mucociliary clearance or production of antimicrobial peptides, mucins, reactive oxygen species, nitrogen species, cytokines, chemokines, and growth factors.^95^ AECs also act as immune barriers via PRRs. AECs express most TLRs, which have a restricted role in recognizing microbial glycoproteins, lipoproteins, and endogenous ligands in mammals.^95^ Signals mediated by C-type lectin receptors involve MyD88 and Toll/interleukin-1 receptor (TIR)-domain-containing adaptor-inducing interferon-β.^95^ C-type lectin receptors recognize mycobacteria and RNA viruses. NOD-like receptors are intracellular PRRs that recognize nucleotides and C-terminal leucine-rich repeats of microbes.^95^ An important component of innate immunity is the release of antimicrobial molecules at the mucosal surface. Intracellular inactive pro-peptides are proteolytically cleaved to active peptides and released in response to microbial stimulation.^96^ However, most of the current studies focus more on inflammatory lung diseases, and the limited research involving lung cancer is focused on immunotherapy.^97^

Given the significant influence of the gut microbiota on mucosal immunity and its potential to modulate remote immune responses in the lungs, it is conceivable that a deeper exploration of the gut-lung axis may unveil novel insights into lung cancer development, prevention, and treatment. Further investigations into the interplay between gut and lung microbiota, mucosal immune responses, and their implications in lung cancer could provide valuable avenues for future research.

## Potential application of the microbiome in the diagnosis and treatment of lung cancer

There is emerging evidence to suggest that the microbiome can be used as a marker for the diagnosis of cancer and may be helpful in the development of personalized treatment for the disease. It is thought that a distinct bronchial microbiome precedes the clinical diagnosis of lung cancer.^98^ Reduced alpha diversity of the lung microbiota, enrichment in specific taxa, and higher bacterial density predict lung cancer status.^99^ Although there are differences in the enrichment of taxa across studies, *Veillonella* and *Capnocytophaga* were found to be enriched in lung cancer and to have good potential for diagnosis of the disease.^21^^,^ ^100^ A microbial-based classifier that uses linear discriminant analysis scores for taxonomic features is capable of predicting lung cancer before diagnosis in patients with no clinical signs of the disease. This classifier uses the linear discriminant analysis result and relative abundance of taxa to construct a score. In this study, a higher abundance of *Streptococcus* and a lower abundance of *Staphylococcus* were associated with a higher risk of lung cancer.^98^

The oral microbiome and gut microbiome are recognized to be risk factors for lung cancer. *Veillonella* is particularly abundant in the oral cavity in patients with lung cancer.^101^ Higher alpha diversity of the oral microbiota is associated with a lower risk of lung cancer. The greater relative abundance of three genera (*Abiotrophia, Lactobacillus*, and *Streptococcus*) and the presence of one genus (*Peptoniphilus*) in the oral cavity are associated with a greater risk of lung cancer.^102^ A predictive model with 13 operational taxonomic unit-based biomarkers, including *Streptococcus infantis, Veillonella dispar*, and others, was found to be highly accurate for the prediction of lung cancer.^103^ Another study found that the oral microbiota was better than the gut microbiota in distinguishing between individuals with and without lung cancer.^101^

In addition to serving as a diagnostic biomarker, the composition of the microbiome has great potential as a predictor of the response to treatment. Microbial-based therapies, such as probiotics and fecal microbiota transplantation, have been extensively studied in the clinical setting. The treatments for lung cancer include surgical resection, radiotherapy, chemotherapy, and immunotherapy, all of which can affect the composition of the microbiota. Chemotherapy-induced intestinal mucositis is attributable in part to dysbiosis of the intestinal microbiota. After radiotherapy and chemotherapy, a unique gut microbial signature linked to higher pro-inflammatory factors, such as IL-6, IL-1β, and tumor necrosis factor-α, is defined in the gut microbiota.^104^, ^105^, ^106^ Microbiota-derived reactive oxygen species can improve the efficacy of chemotherapy, and broad-spectrum antibiotics can eliminate the microbiota, significantly reducing the therapeutic effects of platinum agents.^107^ An intact microbiome is necessary for the pharmacological effects of chemotherapy. However, the prognostic significance of the altered microbiome is unclear.^108^

The immune modulatory effect of the microbiota suggests that it has a role in chemotherapy. Immune checkpoint inhibitors (ICIs) have been approved for use in patients with NSCLC. The efficacy of ICIs appears to be associated with the gut microbiome. Fecal microbiota transplantation from patients with cancer who respond to ICIs into germ-free or antibiotic-treated mice improves the antitumor effect of programmed death-1 (PD-1) blockade, whereas fecal microbiota transplantation from non-responding patients does not.^97^ A diversified gut microbiota composition and specific species, such as *Akkermansia muciniphila* (*A. muciniphila*), *Ruminococcus,* and *Bifidobacterium,* are associated with increased systemic immune tone, thereby improving the clinical response to ICI therapy.^109^ *A. muciniphila* is a Gram-negative human intestinal mucin-degrading bacterium that represents approximately 3–5% of all intestinal bacteria. The abundance of *A. muciniphila* is negatively associated with obesity, type II diabetes, and inflammation.^110^ A higher abundance of *A. muciniphila* and *Enterococcus hirae* in stool samples is positively associated with a favorable clinical outcome of ICI therapy in patients with lung cancer because of their role in increasing the numbers of tumor-infiltrating lymphocytes.^111^

## Limitations of the microbiome: experimental and computational methods

The emergence of microbiome research has significantly enhanced our understanding of the physiological, ecological, and pathological roles of the microbiome. However, despite the large amounts of research, there is a fundamental lack of reproducibility and comparability with regard to the microbiome.^112^^,^^113^

The different findings between studies reflect the systemic biases introduced during sequence-based microbiome research.^114^ The use of different sampling methods highlights regional variation in the lung microbiota; however, systemic biases exist in sample collection methods. General sampling methods used in lung microbiota research include collection by bronchoscopy, lobectomy, and bronchial swab. Homogenized surgical cancer tissues are also used for microbiome research and are mostly indicative of the intracellular microbiome.^115^ BALF collected during bronchoscopy is considered to contain taxa from the upper airway, whereas BALF collected via lobectomy contains more taxa located in the lower region of the respiratory tract and microaspiration of oral secretions.^10^

Analysis of the microbiota using high-throughput sequencing is limited by factors such as the sequencing technology, algorithm, and reference genome used. Differences in workflow and associated bias can also cause variations in results between studies.^112^ Biases resulting from differences in DNA extraction, amplification protocols, and bioinformatics can be comparable to biological differences.^113^ Standardized methods, including DNA extraction and sampling, and appropriate controls are recommended for the microbiome-driven diagnostics, health monitoring services, and interventions.^112^ Some organisms may be unrepresented in databases because of the large number of organisms that have not been cultured or recognized.^116^ Furthermore, genomic data alone cannot determine whether an organism is alive or not. Most bacterial DNA detected in the lungs might come from non-viable bacteria.^20^ To address this issue, complementing genomic data with transcriptomic, proteomic, and metabolomic data sets could be helpful for analyzing gene expression and metabolic data that are more likely to be derived specifically from living cells.^116^

## Conclusion

Although it is widely accepted that lung cancer is associated with altered composition of the lung and gut microbiota, further elucidation is needed to establish a causative role of these microbiota in lung cancer. Emerging evidence suggests that the lung microbiota influences the initiation and progression of lung cancer, but there is still no convincing evidence for a healthy lung microbiota because of confounding factors in population studies and the highly dynamic external environment. In addition to conducting retrospective and prospective cohort studies on the human microbiome and its role in disease at the species level, isolation and functional annotation of specific bacterial species are necessary. Improvements in sequencing technologies and algorithms will help to reveal the complex interactions in the ecosystem and the importance of rare taxa in the initiation and progression of lung cancer. The translation of the outcomes of basic research on the microbiome into diagnostics and therapeutic targets will be research priorities in the upcoming decades.

## Funding

This work was supported by the National Natural Science Foundation of China (Nos. 81773147, 81972198, and 81472695), the Key Research and Development Program of Hunan (No. 2019SK2253), the Strategic Priority Research Program of Central South University (No. ZLXD2017004), and the Natural Science Foundation, Changsha (No. kq2208299).

## Conflicts of interest

The authors declare that they have no known competing financial interests or personal relationships that could have appeared to influence the work reported in this paper.
